# Comparison of family-based association tests in chromosome regions selected by linkage-based confidence intervals

**DOI:** 10.1186/1471-2156-6-S1-S62

**Published:** 2005-12-30

**Authors:** Juan Pablo Lewinger, Sophia SF Lee, Joanna Biernacka, Long Yang Wu, Haijiang Steven Shi, Shelley B Bull

**Affiliations:** 1 Samuel Lunenfeld Research Institute, Mount Sinai Hospital, 600 University Avenue, Toronto, Ontario, Canada M5G 1X5; 2 Department of Public Health Sciences, University of Toronto, 12 Queen's Park Crescent West, Toronto, Ontario, Canada M5S 1A8

## Abstract

We use the Genetic Analysis Workshop 14 simulated data to explore the effectiveness of a two-stage strategy for mapping complex disease loci consisting of an initial genome scan with confidence interval construction for gene location, followed by fine mapping with family-based tests of association on a dense set of single-nucleotide polymorphisms. We considered four types of intervals: the 1-LOD interval, a basic percentile bootstrap confidence interval based on the position of the maximum Zlr score, and asymptotic and bootstrap confidence intervals based on a generalized estimating equations method. For fine mapping we considered two family-based tests of association: a test based on a likelihood ratio statistic and a transmission-disequilibrium-type test implemented in the software FBAT. In two of the simulation replicates, we found that the bootstrap confidence intervals based on the peak Zlr and the 1-LOD support interval always contained the true disease loci and that the likelihood ratio test provided further strong confirmatory evidence of the presence of disease loci in these regions.

## Background

A primary goal of many current human genetic studies is to identify disease susceptibility loci for complex diseases. When there are no obvious candidate genes, a linkage genome scan is usually conducted to select regions for further study. In these regions, further genotyping can be carried out in order to narrow down the possible locations of disease loci using linkage disequilibrium mapping. Given significant genome scan results, the size of the region under a linkage peak on which to concentrate further mapping efforts is not immediately obvious. Confidence intervals (CIs) for the locations of susceptibility genes provide a natural way to determine regions for follow up, yet this is rarely done in practice. Several types of intervals may be considered, such as the 1-LOD score support interval [1] and the generalized estimating equations (GEE) based confidence interval proposed by Liang et al. [2].

Once a region is selected, linkage disequilibrium mapping can be carried out using association methods for family-based designs, which are often preferred over case-control designs because they are immune to potential population stratification. However, standard family-based tests of association such as the transmission-disequilibrium test (TDT) do not utilize all information available in nuclear families, incurring a potential loss of power. Lewinger and Bull [3] recently proposed a likelihood ratio test that makes efficient use of all available information in a nuclear family, including parental phenotypes, genotypes from homozygous parents, and genotypes from both affected and unaffected siblings. Use of this test can lead to substantially increased power [4].

In this paper we use the simulated Genetic Analysis Workshop 14 (GAW14) data to explore the effectiveness of a two-stage strategy for mapping complex disease loci: an initial genome scan with confidence interval construction for disease gene location, followed by fine mapping with family-based association (FBAT) analysis using the likelihood ratio test of Lewinger and Bull [3] and FBAT [5,6].

## Methods

We performed initial multipoint linkage genome scans separately for each of the four populations using Kofendrerd Personality Disorder (KPD) as the phenotype and both the microsatellite and single-nucleotide polymorphism (SNP) marker maps. Analyses of affected sib pairs for Danacaa, Karangar, and Aipotu (the populations with only nuclear family data) were performed with ALLEGRO [7] using the exponential allele-sharing model of Kong and Cox [8] and the S_pairs _scoring function. We identified regions with Zlr peaks exceeding 4.09 corresponding to a genome-wide significance of 2.2 × 10^-5 ^and constructed CIs based on two different estimators of disease gene location: an estimator based on the GEE method proposed by Liang et al. [2] and the simple estimator given by the position of the peak Zlr (LOD) score. The GEE approach, implemented in the GENEFINDER software [9], estimates the location of a disease locus by fitting an expected identity-by-descent (IBD) sharing curve to the observed IBD sharing from a sample of affected sib pairs at a set of linked markers. CIs are computed by relying on the asymptotic normality of the location estimator (using a robust estimate of variance) or by using the basic percentile bootstrap [10]. For the latter, GENEFINDER obtains an estimate of disease gene location for each of 1,000 bootstrap samples of the data and computes the endpoints of a 95% CI by the 2.5 and 97.5 percentiles of the distribution of these 1,000 estimates. Based on the peak Zlr estimator of location we also constructed two types of CIs: a 1-LOD support interval and a bootstrap confidence interval. The 1-LOD support interval is determined by the chromosomal points within 1 LOD unit of the peak LOD score. Although it was originally conceived as a support interval [1] and not as a confidence interval, the 1-LOD interval has approximate 95% coverage when used in the context of parametric linkage analysis. As in the GEE case, the bootstrap interval based on the peak Zlr score was constructed using the basic percentile method with an estimate of disease gene location for each of 500 bootstrap samples of the data. In every case, whenever a CI included the first or last marker of a map, we extended the corresponding upper or lower limit to the end of the chromosome.

Based on the answers, we purchased SNP packets spanning each of the regions containing the disease loci D1-D4. In the populations with significant linkage scan findings, we tested the purchased SNPs for association with KPD status using the test proposed by Lewinger and Bull [3] and FBAT [5,6]. FBAT was used with the additive option that yields the standard TDT if, as is the case in the GAW14 simulated data, there are no missing parental genotypes. Both tests are based on the conditional framework of Rabinowitz and Laird [11]. Let **X **denote all the genotypes and the disease status of all family members in a sample of nuclear families. Let **S **denote the genotypes of the parents only and the disease status of all family members. Under the null hypothesis that the examined marker is *not linked *to any disease predisposing locus, the distribution of the children's genotypes conditional on **S **is independent of the phenotypes and completely determined by Mendelian inheritance. Given any test statistic, randomization of the children's genotypes according to Mendelian probabilities yields a valid test of linkage independent of the distribution of parental genotypes and phenotypes; in particular, tests obtained in this manner are immune to population stratification. The choice of test statistic determines the power of the resulting test. Lewinger and Bull proposed a test statistic based on the standard single locus two-point linkage model with recombination fraction parameter *θ*, penetrance parameters *f*_0_, *f*_1_, *f*_2_, disease allele frequency *p*, marker allele frequency *q*, and a parameter measuring the degree of allelic association between the marker and disease loci, *ψ*. The test statistic is the conditional likelihood ratio based on this model and is given by



where parameters in the numerator are estimated from the conditional likelihood

L(*f*_0_, *f*_1_, *f*_2_, *p*, *q*, *ψ*) = Pr(**S**|**Y**_**a**_; *f*_0_, *f*_1_, *f*_2_, *p*, *q*, *ψ*)

and **Y_a _**is the portion of the phenotypic data on which ascertainment is based. This ensures that the parameters are consistently estimated [12]. The denominator is independent of any of the nuisance parameters because *θ *= 1/2. This statistic uses all available information in a sample of nuclear families, including parental phenotypes, unaffected offspring, and families with homozygous parents. In a series of simulation studies, Lewinger [4] showed that the randomization test based on the conditional likelihood ratio above is more powerful than FBAT in many scenarios. Exact *p*-values for the likelihood ratio test and FBAT/TDT were estimated using an accurate Monte Carlo importance sampling method proposed by Lewinger [4].

## Results

### Genome scans and confidence intervals for gene location

**Figure 1:**
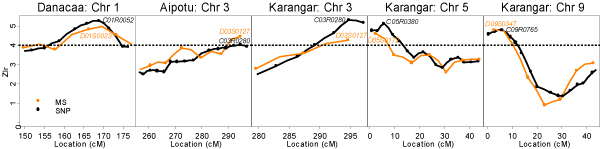
**Genome scan results showing the regions with strong linkage signals in replicate 1**. Danacaa, chromosome 1 (MS between D01S0023 and D01S0024, SNP C01R0052); Aipotu, chromosome 3 (peak at MS marker D03S0127, SNP marker C03R0280; Karangar chromosome 3 (MS D03S0127, SNP C03R0280), chromosome 5 (MS D05S0172, SNP C05R0380), and chromosome 9 (MS D09S0347, SNP C09R0765).

In each of the replicates 1 and 66, linkage genome scans using either of the MS or SNP maps identified regions in chromosomes 1, 3, 5, and 9 where the Zlr score exceeded the 4.09 threshold (Figure 1). In these 4 regions the Zlr score peaked within 5 cM of the true loci, with the scans based on the SNP map tending to peak slightly higher and closer to the disease loci than the scans based on the MS map. In a few cases the Zlr score peaked right on a SNP flanking a disease locus.

**Figure 2:**
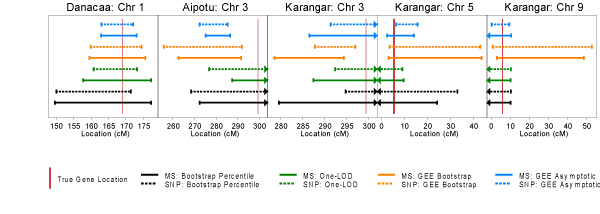
Four types of 95% confidence intervals for disease gene locations using both the microsatellite (MS) and the SNP map in replicate 1.

The 4 confidence intervals obtained from each of the MS and SNP data are shown in Figure 2 for replicate 1. All confidence intervals covered disease locus D1, which is located towards the middle of chromosome 1. For D2-D4, which are near or outside the end of the marker maps, the intervals based on the GEE estimator of location did not perform well, either giving very wide intervals or failing to cover the true disease locus. Similar results were obtained for replicate 66 except that the asymptotic GEE based on MSs did not cover D1 in this case.

Although it might be expected that CIs based on the denser SNP map would be narrower for all types of CIs, we found that this was not always the case (Figure 2). We also expected that CIs based on the GEE estimator of location would be narrower than the Zlr-bootstrap CIs because the GEE method jointly models the IBD sharing pattern at all markers. This method also assumes that there is exactly one disease gene located within the chromosome, which may improve performance when, as in the GAW14 data, this assumption is satisfied. We found this to be the case for D1 but not for D2-D4, which are near ends of chromosomes. Although the asymptotic GEE CIs were always narrower than the bootstrap counterparts, independent simulations have shown that the asymptotic GEE CIs can have less than nominal coverage, particularly when marker density is high [13]. It is remarkable that the simple 1-LOD intervals always covered the true loci and tended to be narrower than all the other CIs. We note however, that the 1-LOD intervals have unknown coverage properties in the context of nonparametric linkage analysis. Further investigation is required to evaluate the performance of different types of CIs for disease gene location.

In the two replicates examined, had we chosen to fine map in the regions spanned by the confidence intervals based on the peak Zlr estimator of location, i.e., the Zlr bootstrap or the 1-LOD, we would have selected SNP packages containing or flanking the true disease loci.

### Fine-mapping analyses of high density SNPs

**Figure 3:**
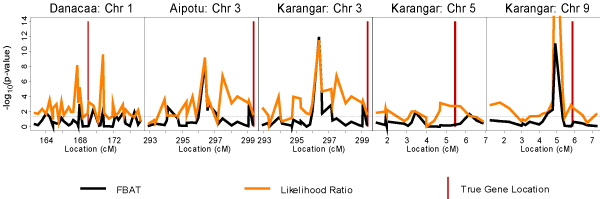
Family-based association test results for replicate 1.

We performed family-based association tests on all the purchased SNP packets including the ones on chromosome 1, which did not have linkage disequilibrium built into the simulation. Figure 3 shows the result for replicate 1. In both replicates, significant results (*α *= 5%, no multiple testing adjustment) were obtained in all of the regions spanned by the SNP packets with very similar patterns of *p*-value "peaks and valleys" for the likelihood ratio test and FBAT. However, the likelihood ratio test gave smaller *p*-values than the FBAT/TDT for most SNPs and in particular the SNPs flanking disease loci gave significant results with the likelihood ratio test but were not found to be associated by FBAT/TDT. This is consistent with simulations that showed that the likelihood ratio test can be more powerful than the TDT for many scenarios [4]. We were surprised to obtain strongly significant family-based association results for chromosome 1 in both replicates given that no linkage disequilibrium was simulated in this region and weak results for chromosome 5 in replicate 1 which was simulated with linkage disequilibrium.

When compared to the Zlr linkage peaks, the FBATs did not succeed in "getting closer" to the true disease loci, but to gauge the full potential narrowing of chromosomal regions with FBAT methods, some form of interval estimate would be required. This is beyond the scope of this study. However, the likelihood ratio test provided strong confirmatory evidence of the presence of disease loci.

It is noteworthy that we found considerable discrepancies between exact *p*-values and the *p*-values obtained using an asymptotic normal approximation (results not shown), particularly for the likelihood ratio test. This shows the importance of accurate computation of *p*-values.

## Conclusion

The initial genome scan identified the four main disease loci in replicates 1 and 66 of the GAW14 simulated data. The regions for follow-up determined by the bootstrap CI based on the peak Zlr and and the 1-LOD support always contained the true disease loci in these two replicates and the likelihood ratio test of Lewinger and Bull [3] provided strong confirmatory evidence of the presence of disease loci in these regions. To the extent that the simulated data captures the complexities of multifactorial diseases, we believe that, as implemented here, this type of two-stage strategy holds promise for finding real disease genes.

## Abbreviations

CI: Confidence interval

FBAT: Family-based association test

GAW14: Genetic Analysis Workshop 14

GEE: Generalized estimating equations

IBD: Identity-by-descent

KPD: Kofendrerd Personality Disorder

SNP: Single-nucleotide polymorphism

## Authors' contributions

JPL designed the study, conducted the family-based association analyses, and drafted the manuscript. SSFL assisted in the design of the study, computed the 1-LOD and bootstrap Zlr confidence intervals, and prepared the figures. JB computed the GEE confidence intervals and assisted in designing the study and drafting the manuscript. LYW bootstrapped families to obtain resampling estimates of location. HSS conducted the genome scans and assisted with the association analyses. SBB assisted in designing the study and revising the manuscript. All authors read and approved the final manuscript.

## References

[B1] Terwilliger JD, Ott J (1994). Handbook of Human Genetic Linkage.

[B2] Liang KY, Chiu YF, Beaty TH (2001). A robust identity-by-descent procedure using affected sib-pairs: multipoint mapping for complex diseases. Hum Hered.

[B3] Lewinger JP, Bull SB (2003). A powerful test of linkage in the presence of association for nuclear families with arbitrary patterns of missing information [abstract]. Am J Hum Genet.

[B4] Lewinger JP (2004). Family-based nonparametric tests of linkage and association. PhD thesis.

[B5] Laird N, Horvath S, Xu X (2000). Implementing a unified approach to family based tests of association. Genet Epidemiol.

[B6] FBAT. Family Based Association Testing Software. http://www.biostat.harvard.edu/~fbat.

[B7] Gudbjartsson DF, Jonasson K, Frigge ML, Kong A (2000). Allegro, a new computer program for multipoint linkage analysis. Nat Genet.

[B8] Kong A, Cox NJ (1997). Allele-sharing models: LOD scores and accurate linkage tests. Am J Hum Genet.

[B9] GENEFINDER. http://www.biostat.jhsph.edu/~wmchen/gf.html.

[B10] Davison AC, Hinkley DV (1997). Bootstrap Methods and Their Applications.

[B11] Rabinowitz D, Laird N (2000). A unified approach to adjusting association tests for population admixture with arbitrary pedigree structure and arbitrary misssing marker information. Hum Hered.

[B12] Ewens WC, Shute NC (1986). A resolution of the ascertainment sampling problem. I. Theory. Theor Popul Biol.

[B13] Biernacka JM (2005). Statistical methods for studying two linked disease genes. PhD thesis.

